# Predictive Utility of Mortality by Aging Measures at Different Hierarchical Levels and the Response to Modifiable Life Style Factors: Implications for Geroprotective Programs

**DOI:** 10.3389/fmed.2022.831260

**Published:** 2022-04-22

**Authors:** Jingyun Zhang, Xingqi Cao, Chen Chen, Liu He, Ziyang Ren, Junhua Xiao, Liyuan Han, Xifeng Wu, Zuyun Liu

**Affiliations:** ^1^Center for Clinical Big Data and Analytics of the Second Affiliated Hospital and Department of Big Data in Health Science School of Public Health, Zhejiang University School of Medicine, Hangzhou, China; ^2^National Institute of Environmental and Health, Chinese Center for Disease Control and Prevention, Beijing, China; ^3^College of Chemistry, Chemical Engineering and Biotechnology, Donghua University, Shanghai, China; ^4^Department of Global Health, Ningbo Institute of Life and Health Industry, University of Chinese Academy of Sciences, Ningbo, China; ^5^Hwa Mei Hospital, University of Chinese Academy of Sciences, Ningbo, China

**Keywords:** aging, frailty, telomere shortening, mortality, life style

## Abstract

**Background:**

Aging, as a multi-dimensional process, can be measured at different hierarchical levels including biological, phenotypic, and functional levels. The aims of this study were to: (1) compare the predictive utility of mortality by three aging measures at three hierarchical levels; (2) develop a composite aging measure that integrated aging measures at different hierarchical levels; and (3) evaluate the response of these aging measures to modifiable life style factors.

**Methods:**

Data from National Health and Nutrition Examination Survey 1999–2002 were used. Three aging measures included telomere length (TL, biological level), Phenotypic Age (PA, phenotypic level), and frailty index (FI, functional level). Mortality information was collected until December 2015. Cox proportional hazards regression and multiple linear regression models were performed.

**Results:**

A total of 3,249 participants (20–84 years) were included. Both accelerations (accounting for chronological age) of PA and FI were significantly associated with mortality, with HRs of 1.67 [95% confidence interval (CI) = 1.41–1.98] and 1.59 (95% CI = 1.35–1.87), respectively, while that of TL showed non-significant associations. We thus developed a new composite aging measure (named PC1) integrating the accelerations of PA and FI, and demonstrated its better predictive utility relative to each single aging measure. PC1, as well as the accelerations of PA and FI, were responsive to several life style factors including smoking status, body mass index, alcohol consumption, and leisure-time physical activity.

**Conclusion:**

This study demonstrates that both phenotypic (i.e., PA) and functional (i.e., FI) aging measures can capture mortality risk and respond to modifiable life style factors, despite their inherent differences. Furthermore, the PC1 that integrated phenotypic and functional aging measures outperforms in predicting mortality risk in comparison with each single aging measure, and strongly responds to modifiable life style factors. The findings suggest the complementary of aging measures at different hierarchical levels and highlight the potential of life style-targeted interventions as geroprotective programs.

## Introduction

Aging is a critical risk factor for many chronic diseases. As a comprehensive and multi-dimensional process, aging could be measured at different hierarchical levels, including biological, phenotypic and functional levels (1). Biological aging measures focus on changes at the molecular, cellar, and intracellular levels, such as telomere length (TL) and DNA methylation clocks (1–3). Phenotypic aging measures include composite indexes derived from multi-system clinical chemistry biomarkers, such as Phenotypic Age (PA) (4), reflecting changes in body composition, homeostatic mechanisms, energetics, and brain health over time. Functional aging measures include composite indexes derived from different functional aspects (e.g., cognitive and physical function). Frailty index (FI) is a widely used functional aging measure that integrates deficits across multiple functional domains (5–7). These aging measures are conceptually different; however, direct comparative analyses of their predictive utility of mortality risk are limited. To the best of our knowledge, only one study based on adults > 50 years in Sweden compared aging measures at three hierarchical levels (8). Since aging starts early in life (9), it remains unclear how these aging measures behaves in terms of mortality prediction among a general population with younger, middle-aged, and older adults. It is also of interest to examine whether integrating two or more aging measures at different hierarchical levels would provide a more informative one, which is valuable in geroprotective programs where these aging measures serve as endpoints to help with assessing the effectiveness of interventions.

One important feature of qualifying aging measures includes effective responsiveness to interventions (10). This feature has been rarely emphasized in previous work whereas it is the key to the application of aging measures in clinical settings. Life styles such as smoking and physical activity are modifiable factors and have been demonstrated to be associated with individual aging measures such as TL (11) and FI (12, 13). However, few studies have simultaneously evaluated the response of aging measures at different hierarchical levels to modifiable life style factors in the same population.

Using data from the National Health and Nutrition Examination Survey (NHANES) 1999–2002, including three aging measures at three hierarchical levels (i.e., TL, PA, and FI), this study aimed to (1) compare the predictive utility of mortality risk by three aging measures at three hierarchical levels; (2) develop a new composite aging measure that integrated aging measures at different hierarchical levels; and (3) evaluate the response of these aging measures to modifiable life style factors.

## Materials and Methods

### Study Population

NHANES is an ongoing program conducted by the National Center for Health Statistics of the Centers for Disease Control and Prevention in the United States. NHANES began in the early 1960s and focuses on the health and nutritional status of adults and children in the United States. Since 1999, NHANES has become a continuous program that collects a wide range of health-related data via interview, examination, and laboratory tests in counties across the country biennially (4). NHANES is approved by the National Center for Health Statistics Research Ethics Review Board (Protocol #98-12), and all participants provided informed consent. In this study, we included participants with TL data and complete information to calculate PA and FI. In total, 3,249 of 9,882 participants aged from 20 to 84 years in NHANES 1999–2002 were included. NHANES data are publicly available (https://www.cdc.gov/nchs/nhanes/index.htm). The analytic roadmap of this study is shown in Supplementary Figure 1.

### Measurements

#### TL and Acceleration of TL (TL.Accel)

In NHANES, TL assay was performed in the laboratory of Dr. Elizabeth Blackburn at the University of California, San Francisco, using the quantitative polymerase chain reaction (PCR) method to measure TL relative to standard reference DNA (T/S ratio) based on blood samples (14, 15). Each sample was assayed three times on three different days. The mean of the T/S ratio was used to represent TL and details of laboratory methods are described at the official website of NHANES (https://wwwn.cdc.gov/Nchs/Nhanes/1999-2000/TELO_A.htm). To eliminate the effect of chronological age (CA) on TL, we calculated a new index, TL.Accel, defined as residual from a linear model when regressing TL on CA. TL.Accel was classified into normal (TL.Accel ≥ 0, indicating that a participant's TL is equal or longer than expected based on his/her CA) or shorter (TL.Accel < 0, indicating that a participant's TL is shorter than expected based on his/her CA).

#### PA and Acceleration of PA (PA.Accel)

PA was first developed based on NHANES III (4, 16). In brief, PA was derived from CA and 9 biomarkers including albumin, creatinine, glucose, (log) C-reactive protein, lymphocyte percent, mean cell volume, red cell distribution width, alkaline phosphatase, and white blood cell count. As done to TL, we calculated PA.Accel, defined as residual from a linear model when regressing PA on CA. PA.Accel represents phenotypic aging after accounting for CA, i.e., a participant is phenotypically older (younger) if his/her PA.Accel > 0 (< 0) than expected based on his/her CA (4, 16).

#### FI and Acceleration of FI (FI.Accel)

FI integrates 36-item deficits (Supplementary Table 1) including comorbidities, activities of daily living, physical tasks, cognition, and performance testing (17). FI was calculated as a ratio of the number of deficits in a participant out of the total possible deficits considered, with a range of 0–1, and the higher score indicates the frailer a participant was. FI.Accel was defined as residual from a linear model when regressing FI on CA. FI.Accel was used as a categorical variable, and divided into frail (FI.Accel > 0) or robust (FI.Accel ≤ 0).

#### Mortality

Mortality follow-up was based on linked data from records taken from the National Death Index (NDI) through December 31, 2015, provided by the Centers for Disease Control and Prevention (18). Survival time was calculated as months from the date of interview to the date of death or the end of follow-up, whichever came first.

#### Covariates

Demographic factors (CA, gender, ethnicity, and education level), body mass index (BMI), and life style factors [i.e., smoking status, binge drinking status, alcohol consumption, leisure-time physical activity level (PAQ), and health eating index-2010 (HEI-2010) (19)] were included as covariates. Ethnicity was grouped as non-Hispanic white, non-Hispanic black, Hispanic, and others. Education level was grouped as less than high school (<HS), HS/general educational development (HS/GED), having attended college but not receiving at least a bachelor's degree (some college), and having a bachelor's degree or higher (college). BMI was grouped as underweight (BMI < 18.5 kg/m^2^), normal (18.5 kg/m^2^ ≤ BMI < 25 kg/m^2^), overweight (25 kg/m^2^ ≤ BMI < 30 kg/m^2^), and obese (BMI ≥ 30 kg/m^2^). Smoking status was grouped as never smoker, former smoker, and current smoker. Alcohol consumption was grouped as never drinker (never drinking or didn't drink in the past year), low to moderate drinker (drinks <3 times per month), and heavy drinker (drinks at least one time per week). PAQ was grouped as low (<one time per week), moderate (1–2 times per week), and heavy (≥3 times per week). HEI-2010 was grouped by tertiles (Tertile 1, 2, and 3).

### Statistical Analyses

The basic characteristics are presented as mean ± standard deviation (SD) and number (percentage) for continuous and categorical variables, respectively.

To assess the predictive utilities for all-cause mortality of three aging measures, survival analysis was conducted. Kaplan–Meier (K-M) curves were plotted and log-rank tests were conducted. Meanwhile, Cox proportional hazards regression was performed based on three models: model 1 was a crude model; model 2 adjusted for CA and gender; and model 3 additionally adjusted for ethnicity, education level, smoking status, alcohol consumption, binge drinking status, BMI, PAQ, and HEI-2010 based on model 2. Hazard ratio (HR) and 95% confidence intervals (95% CI) were documented. Next, time-dependent receiver operating characteristic (ROC) curves (20) were applied to evaluate the predictive utility of different aging measures using model 2 and model 3. Three indices of predictive utility [i.e., area under the curve (AUC), integrated discrimination improvement (IDI), and continuous net reclassification improvement (NRI) (21)] for each of three aging measures were calculated, in comparison to those of the basic model with CA and gender only.

Since two of the three aging measures (PA.Accel and FI.Accel) outperformed relative to TL.Accel, we next tried to develop a new composite aging measure with better predictive utility by integrating aging measures at different hierarchical levels. Principal component analysis (PCA) was applied to PA.Accel and FI.Accel, and the first principal component (PC1) was defined as a new composite aging measure. We then performed the same analyses (i.e., K-M curves and Cox proportional hazards regression) to assess the predictive utility for all-cause mortality of PC1.

We applied linear regression to examine the responses to the life style factors (i.e., smoking status, BMI, alcohol consumption, binge drinking status, PAQ, and HEI-2010) of PA.Accel, FI.Accel, and PC1, the three showing significant predictive utilities of mortality in the previous analysis. Because PC1 was scaled, PA.Accel and FI.Accel were also scaled for comparability. We adjusted for CA and gender, and documented regression coefficients and 95% CI in these associations.

We performed several sensitivity analyses. Due to the wide age range of study population, we tested whether the associations of three aging measures and the new composite age measure with mortality differed by age groups. Moreover, we also estimated whether the responses of PA.Accel, FI.Accel, and PC1 to the life style factors differed by age groups. Additionally, the durations of the exposition to life style risk factors may have potential impact on aging; thus, we estimated the association of smoking duration (the only available variable in NHANES) with PA.Accel, FI.Accel, and PC1, for previous and current smokers.

All analyses were conducted via R (version 4.0.3, 2020-10-10) and a two-sided *p* < 0.05 was considered to be statistically significant.

## Results

### Basic Characteristics of Study Participants

The basic characteristics of 3,249 participants are shown in Table 1. The mean CA of 3,249 participants was 48.4 ± 17.8 years and around a third of them were old adults (≥60 years). Around half of the participants were females (50.8%). The proportions of non-Hispanic white, non-Hispanic black, and Hispanic were 50.9, 15.7, and 30.6%, respectively. More than half of the participants didn't go to college and only 18.2% received a bachelor's degree or over. Half of the participants were never smokers, and 22.4% were current smokers. The proportions of participants at different alcohol consumption levels were similar. Around 13% of the participants reported being binge drinkers. Only 1.2% of participants were underweight and around 31% had normal weight. More than half reported performing physical activity < 1 time per week. The mean HEI-2010 of the three tertiles group were 31.5, 45.7, and 62.3, respectively.

**Table 1:** Characteristics of the study participants, NHANES 1999–2002.

**Characteristics**	**No. (%) or mean ±SD**
All	3,249
Chronological age, y	48.4 ± 17.8
Young- and middle-aged adults (20–59 years)	2,206 (67.9)
Older adults (60–84 years)	1,043 (32.1)
**Gender**	
Female	1,649 (50.8)
Male	1,600 (49.2)
**Ethnicity**	
Non-Hispanic white	1,653 (50.9)
Non-Hispanic black	510 (15.7)
Hispanic	995 (30.6)
Others	91 (2.8)
**Education^a^**	
< HS	1,056 (32.5)
HS/GED	742 (22.9)
Some college	858 (26.4)
College	589 (18.2)
**Smoking status**	
Never smoker	1,635 (50.4)
Former smoker	882 (27.2)
Current smoker	727 (22.4)
**BMI^b^**	
Normal	990 (30.9)
Underweight	40 (1.2)
Overweight	1,177 (36.7)
Obese	998 (31.1)
**Alcohol consumption^c^**	
Never drinker	1,042 (33.2)
Low to moderate drinker	1,096 (34.9)
Heavy drinker	1,002(31.9)
**Binge drinking status**	
Yes	415 (12.8)
No	2,834 (87.2)
**PAQ**	
<1 time/week	1,857 (57.2)
1–2 times/week	1,099 (33.9)
≥3 times/week	289 (8.9)
**HEI-2010**	
Tertile 1	31.5 ± 5.4
Tertile 2	45.7 ± 3.8
Tertile 3	62.3 ± 8.0
**Three aging measures**	
Frailty index	0.11 ± 0.09
Phenotypic age, y	41.56 ± 19.45
Telomere length	1.02 ± 0.26

*NHANES, the National Health and Nutrition Examination Survey; SD, standard deviation; HS, high school; GED, general educational development; BMI, body mass index; PAQ, leisure time physical activity level; HEI, health eating index*.

*Percentages may not sum to 100 because of rounding. There were missing data on education (n = 4), smoking status (n = 5), drinking status (n = 109), BMI (n = 44), PAQ (n = 4), and HEI-2010 (n = 40)*.

a *Education levels included less than HS (<HS), HS/GED, having attended college but not receiving at least a bachelor's degree (some college), and having a bachelor's degree or higher (college)*.

b *Underweight was defined as BMI < 18.5 kg/m^2^; normal was defined as 18.5 ≤ BMI < 25.0 kg/m^2^; overweight was defined as 25.0 ≤ BMI < 30.0 kg/m^2^; and obese was defined as BMI ≥ 30.0 kg/m^2^*.

c *Alcohol consumption was defined as never drinker (never drinking or didn't drink in past year), low to moderate drinker (drinks <3 times per month), and heavy drinker (drinks at least one time per week)*.

### Were Three Aging Measures Correlated to CA?

**Figure 1:**
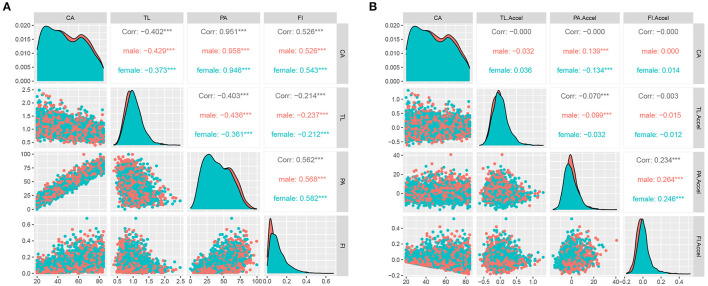
Correlations between three aging measures and chronological age. CA, chronological age; TL, telomere length; PA, Phenotypic age; FI, frailty index; TL.Accel, PA.Accel and FI.Accel represent residuals from linear models when regressing TL, PA, and FI on CA, respectively. ***p < 0.001. **(A,B)** represent correlations before and after adjustments of chronological age, respectively.

As shown in Figure 1A, all three aging measures significantly were correlated to CA. Among them, shorter TL was correlated to older CA with a Pearson correlation coefficient of −0.40, while the other two aging measures were positively correlated to CA. Figure 1B illustrates the correlations after eliminating the effects of CA on aging measures by linear regression.

### Did Three Aging Measures Predict All-Cause Mortality?

**Figure 2:**
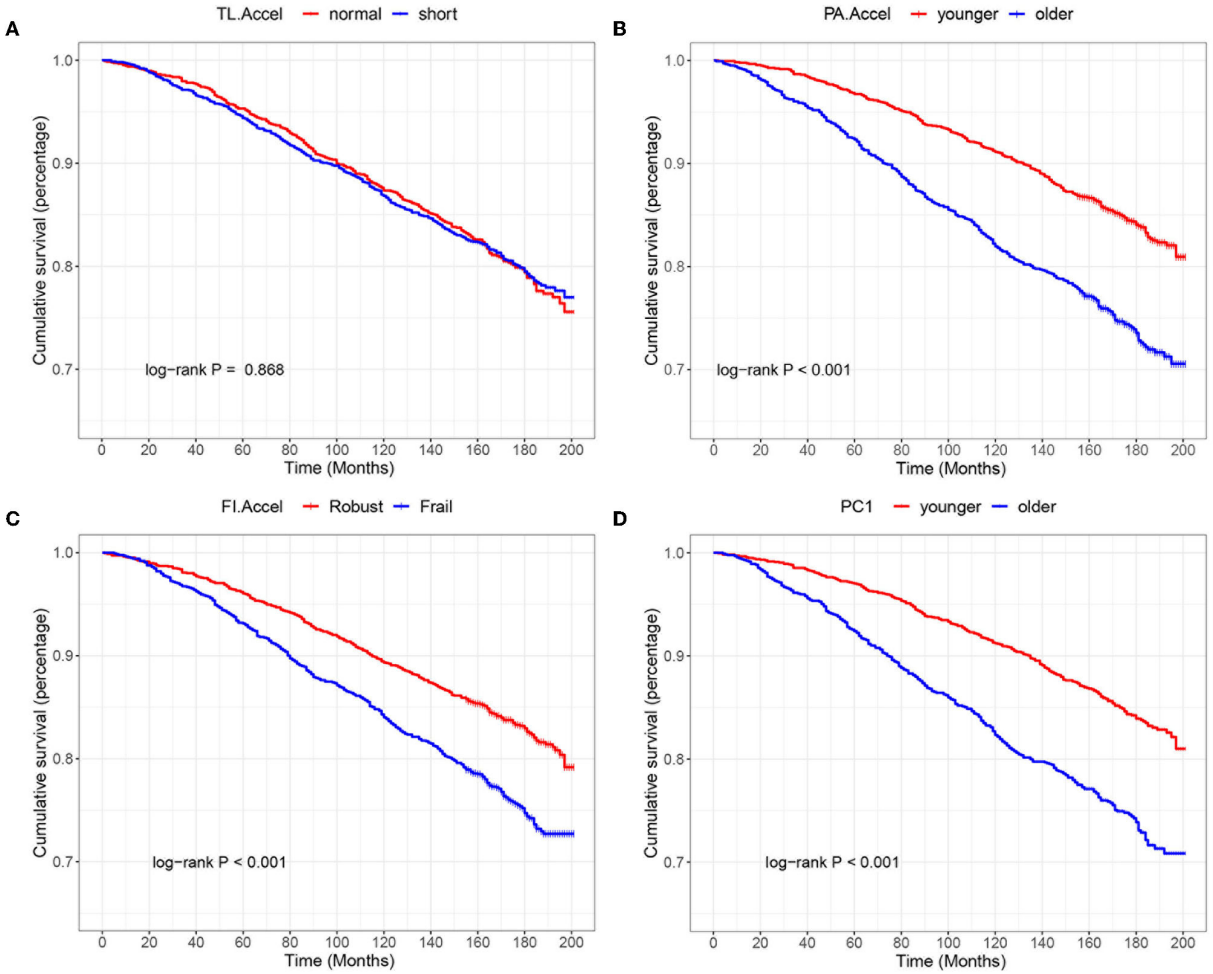
K-M curves of different aging measures for predicting all-cause mortality. TL.Accel, PA.Accel and FI.Accel represent residuals from linear models when regressing telomere length, Phenotypic age, and frailty index on chronological age, respectively. PC1 is the first principal component of PA.Accel and FI.Accel through the principal component analysis. **(A–D)** represent K-M curves of TL.Accel, PA.Accel, FI.Accel and PC1 for predicting all-cause mortality, respectively.

Figures 2A–C presents the associations of the three aging measures with mortality. We found that PA.Accel (log-rank *p* < 0.001) and FI.Accel (log-rank *p* < 0.001), but not TL.Accel (log-rank *p* = 0.868), could identify participants at different risks of death. The similar results implied by Cox regression are shown in Table 2. According to the crude model (model 1), compared to phenotypically younger participants (PA.Accel < 0), phenotypically older participants (PA.Accel ≥ 0) had a 79% increase in mortality risk (HR = 1.79, 95% CI = 1.54–2.09). Similarly, compared to robust participants (FI.Accel ≥ 0), frail ones (FI.Accel < 0) had a 52% increase in mortality risk (HR = 1.52, 95% CI = 1.31–1.77). However, TL.Accel was found not to be significantly associated with mortality risk based on Cox regression (*p* = 0.868). After adjusting for covariates, these associations did not change substantially (models 2 and 3).

**Table 2:** Associations of three aging measures with mortality.

	**No. of death (%)**	**Model 1**		**Model 2**		**Model 3**	
		**HR (95% CI)**	***p***	**HR (95% CI)**	***p***	**HR (95% CI)**	***p***
**TL.Accel**							
Short	301 (20.46)	Ref	–	Ref	–	Ref	–
Normal	362 (20.36)	0.99 (0.85–1.15)	0.868	1.00 (0.86–1.17)	0.992	0.97 (0.82–1.14)	0.711
**PA.Accel**							
Younger	291 (16.04)	Ref	–	Ref	–	Ref	–
Older	372 (25.92)	1.79 (1.54–2.09)	**<0.001**	1.85 (1.58–2.16)	**<0.001**	1.67 (1.41–1.98)	**<0.001**
**FI.Accel**							
Robust	321 (17.19)	Ref	–	Ref	–	Ref	–
Frail	342 (24.75)	1.52 (1.31–1.77)	**<0.001**	1.62 (1.38–1.88)	**<0.001**	1.59 (1.35–1.87)	**<0.001**
**PC1**							
Younger	313 (16.60)	Ref	–	Ref	–	Ref	–
Older	350 (25.68)	1.80 (1.54–2.11)	**<0.001**	1.85 (1.58–2.17)	**<0.001**	1.79 (1.51–2.12)	**<0.001**

*HR, hazard ratio; TL.Accel, PA.Accel, and FI.Accel represent residuals from linear models when regressing telomere length, Phenotypic age, and frailty index on chronological age, respectively; PC1, the first principal component of PA.Accel and FI.Accel through the principal component analysis*.

*Model 1 was a crude model; model 2 adjusted for chronological age and gender; model 3 further adjusted for ethnicity, education level, body mass index, smoking status, binge drinking status, alcohol consumption, leisure time physical activity level, and health eating index based on model 2. The bold values represent that the tests were statistically significant with two-tailed p < 0.05*.

### Did Aging Measures Show Additional Predictive Utilities Than CA and Gender?

**Figure 3:**
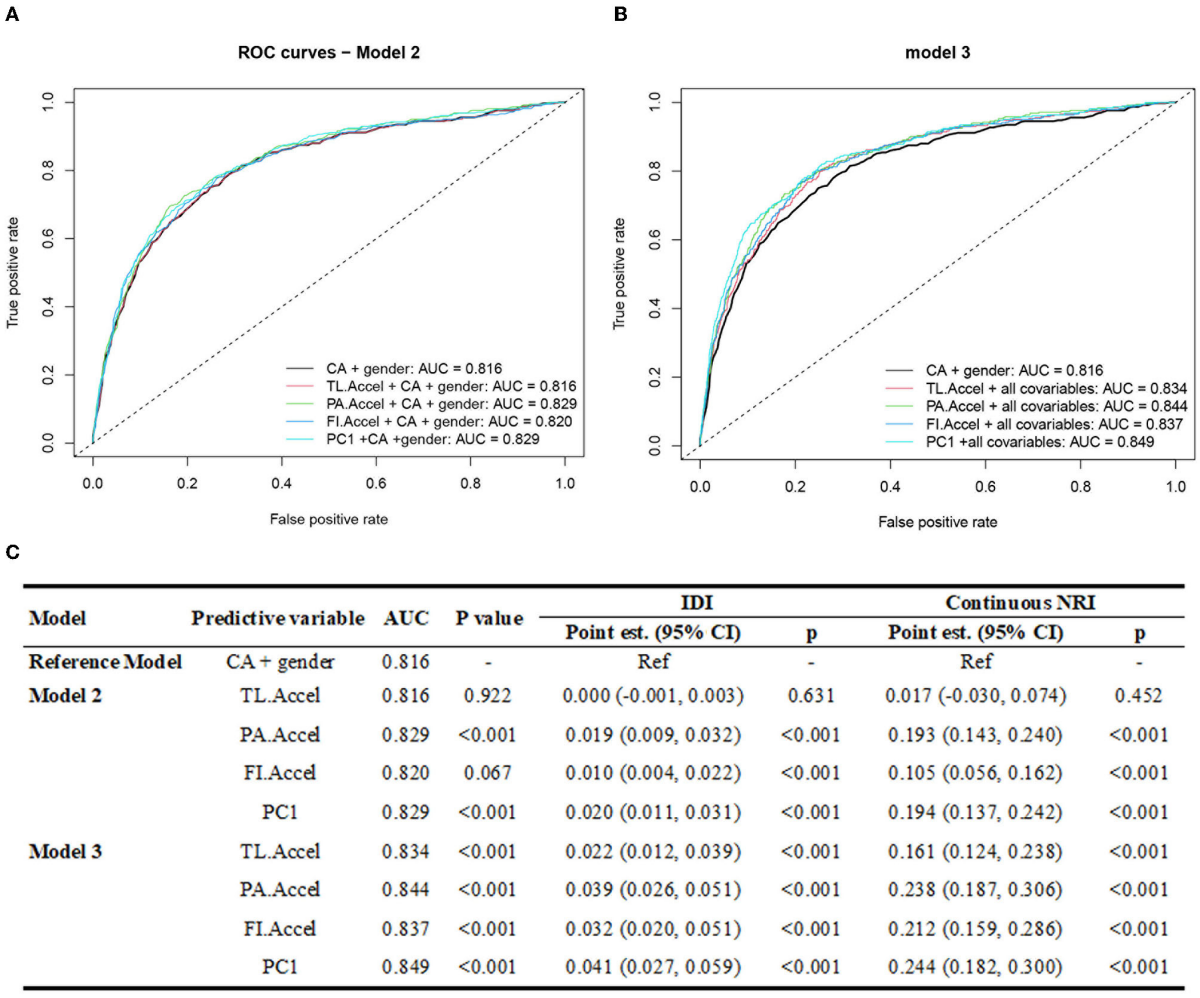
The predictive performance of different aging measures. CA, chronological age; TL, telomere length; PA, phenotypic age; FI, Frailty index; TL.Accel, PA.Accel and FI.Accel represent residuals from linear models when regressing telomere length, Phenotypic age and frailty index on CA, respectively; PC1, the first principal component of PA.Accel and FI.Accel through the principal component analysis; AUC, area under the curve; IDI, integrated discrimination improvement; NRI, net reclassification improvement; est., estimation. Model 2 adjusted for CA and gender; Model 3 further adjusted for ethnicity, body mass index, education level, smoking status, alcohol consumption, binge drinking status, leisure time physical activity level, and health eating index based on Model 2. **(A)** and **(B)** represent the ROC curves of different aging measures based on model 2 and 3, respectively. **(C)** shows the AUC, IDI and NRI of each aging measure and the comparison to reference model.

Figures 3A,B exhibits the ROC curves for predicting mortality by different aging measures.

Compared to the basic model (only CA and gender were included, AUC = 0.816), the model with PA.Accel or FI.Accel had higher predictive utility, evidenced by significantly increased AUC (PA.Accel: 0.829, *p* < 0.001; FI.Accel: 0.820, *p* = 0.067 in model 2), IDI (PA.Accel: 0.019, *p* < 0.001; FI.Accel: 0.010, *p* < 0.001 in model 2), and continuous NRI (PA.Accel: 0.193, *p* < 0.001; FI.Accel: 0.105, *p* < 0.001 in model 2). We did not observe that TL.Accel added significantly predictive utility. When adjusting for more covariates in the models (i.e., model 3), we observed similar patterns.

### Can We Develop a New Composite Aging Measure?

Due to the inherent difference shared by aging measures at different hierarchical levels and the better predictive utility of PA.Accel and FI.Accel (relative to TL.Accel), we asked that whether we could develop a new composite aging measure with a better predictive utility by integrating aging measures at different hierarchical levels. Thus, PCA was applied to PA.Accel and FI.Accel and the scatter plot of PCA is shown in Supplementary Figure 2. We found that PC1 accounted for 61.70% of the total variance and can be calculated as follows:


(1) $$\begin{array}{r} PC1=0.707\;\times PA.Accel+0.707\;\times \;FI.Accel \end{array}$$

We then calculated PC1 for each participant. As shown in Figure 2D, PC1 could identify participants at different risks of death (log-rank *p* < 0.001). Moreover, we found that PC1 outperformed each single aging measure (Figure 3C) with larger AUC (0.829, *p* < 0.001, model 2), and greater increases of IDI (0.020, *p* < 0.001, model 2) and NRI (0.194, *p* < 0.001, model 2) compared to the basic model, and the pattern was more obvious in model 3.

### Are These Aging Measures Responsive to Modifiable Life Style Factors?

**Figure 4:**
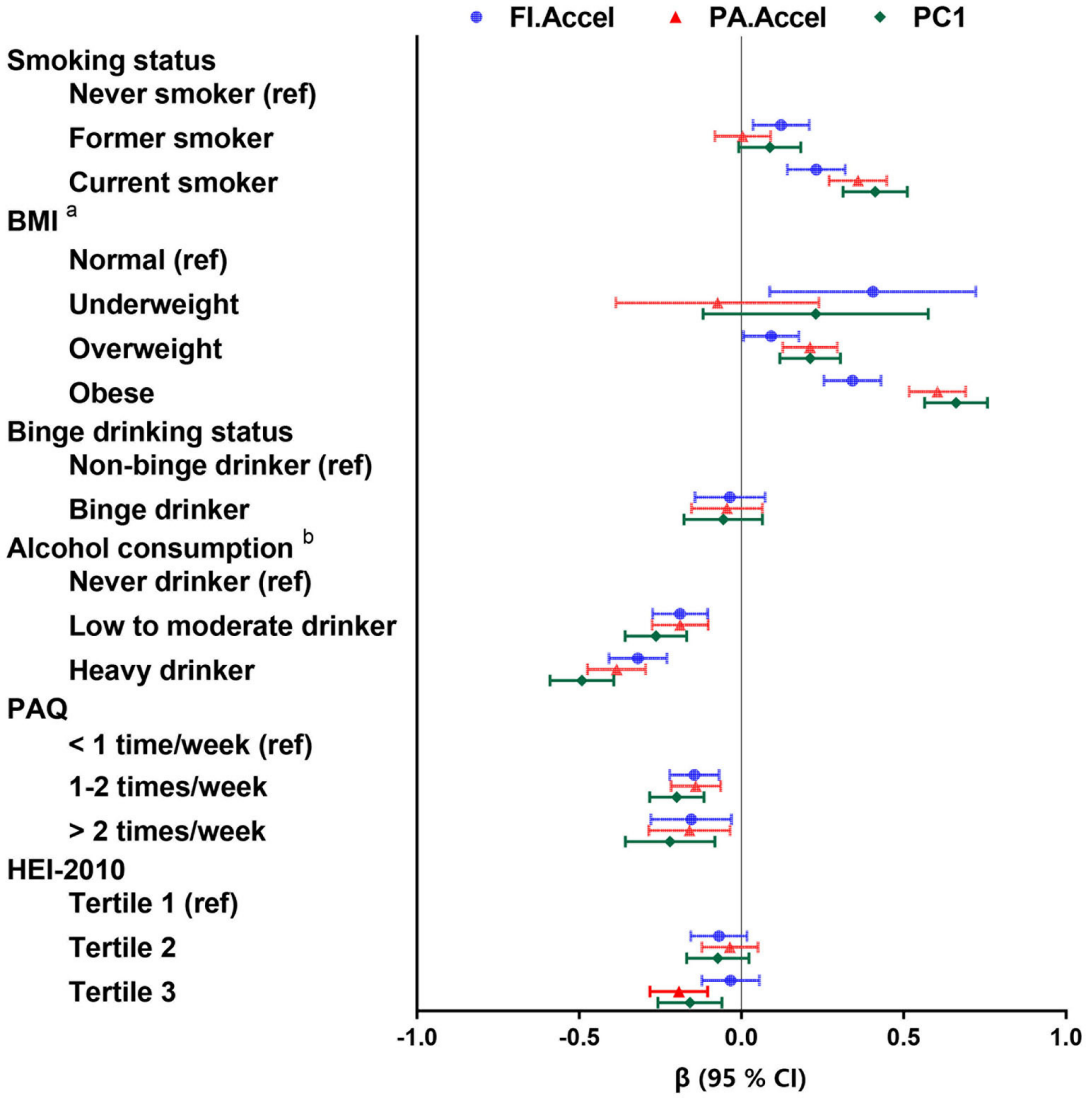
The responses of different aging measures to modifiable life style factors. Coefficients (β) and 95% confidence intervals (CI) were calculated via linear regression adjusted for chronological age and gender. PA.Accel and FI.Accel represent residuals from linear models when regressing telomere length, Phenotypic age and frailty index on chronological age, respectively. PC1, the first principal component of PA.Accel and FI.Accel through the principal component analysis; BMI, body mass index; PAQ, leisure time physical activity level; HEI, health eating index. ^a^Alcohol consumption was defined as never drinker (never drinking or didn't drink in past year), low to moderate drinker (drinks <3 times per month), and heavy drinker (drinks at least one time per week). ^b^Underweight was defined as BMI < 18.5 kg/m^2^; normal was defined as 18.5 ≤ BMI < 25.0 kg/m^2^; overweight was defined as 25.0 ≤ BMI < 30.0 kg/m^2^; and obese was defined as BMI ≥ 30.0 kg/m^2^.

Figure 4 presents results from linear regression to examine the association of modifiable life style factors (i.e., smoking status, BMI, binge drinking status, alcohol consumption, PAQ, and HEI-2010) of PA.Accel, FI.Accel and PC1, the three showing significant predictive utilities of mortality in the previous analysis. Overall, PA.Accel, FI.Accel, and PC1 were responsive to smoking status, BMI, alcohol consumption, and PAQ. For instance, compared to never smokers, current smokers had a significantly higher level of PA.Accel (β = 0.36, *p* < 0.001) and FI.Accel (β = 0.23, *p* < 0.001). Interestingly, relative to PA.Accel and FI.Accel, PC1 showed stronger responses to almost all modifiable life style factors (except for HEI-2010), with the largest absolute values of regression coefficients in these associations (Supplementary Table 2), indicating that the new composite aging measure might be more sensitive to modifiable life style factors.

### Sensitivity Analysis

Supplementary Table 3 shows the associations of the three aging measures and PC1 with mortality by age groups. After adjusting for all covariates (model 3), both single aging measure (i.e., PA.Accel and FI.Accle) and the new composited aging measure (i.e., PC1) showed predictive utilities of mortality risk in different age groups. However, compared to short TL, normal TL (HR = 0.70, 95% CI = 0.51–0.97) was a protective factor of mortality among young- and middle-aged adults (20–59 years).

Supplementary Table 4 shows the responses of different aging measures to modifiable life style factors by age groups. Among young- and middle-aged adults (20–59 years), aging measures responded to modifiable life style factors, as observed in the total population. However, among older adults (60–84 years), fewer significant associations were observed. For instance, FI.Accel and PC1 didn't respond to diet quality (HEI-2010) anymore, but they were both responsive to HEI-2010 among young- and middle-aged adults. Furthermore, PA.Accel didn't respond to smoking status anymore, but PC1 remained a significant response to smoking status.

Supplementary Table 5 shows that for previous smokers, longer durations of smoking were associated with higher level of PA.Accel and PC1. No above associations were observed for current smokers.

## Discussion

Based on the unique data from US NHANES, this study demonstrated that both PA and FI, but not TL, was significantly predictive of all-cause mortality. Building on the better performance of PA and FI, we integrated them to develop a new composite aging measure, which has been demonstrated to be predictive of mortality risk as well, even better than each single aging measure. Finally, we demonstrated that PA and FI, as well as the new composite aging measure, were responsive to some modifiable life style factors, including smoking status, alcohol consumption, and PAQ. The findings, for the first time, provide a full picture of the predictive utility of mortality risk by three aging measures at three hierarchical levels and the response to modifiable life style factors, with important implications for geroprotective programs.

The findings of the positive associations of PA and FI with all-cause mortality risk are consistent with previous studies (4, 22–25). To date, the association of TL and mortality remains less conclusive in epidemiological studies (25–28), and the discrepancy may be partly explained by the differences among the study populations, and methods to measure TL (29). Two studies based on the Dunedin birth cohort (25) and the National Health and Nutrition Examination Survey (NHANES) (27), respectively, considered TL and PA, and reported that TL was not consistently associated with multiple health span-related characteristics as compared to PA. However, FI was not considered in these two studies. The current study fills this knowledge gap by simultaneously evaluating the predictive utility of mortality risk by three aging measures at three hierarchical levels. The differences observed confirm that these aging measures did not necessarily reflect the same aging processes, as originally proposed by Ferrucci et al. (1).

The increased predictive utility by PC1 relative to each single aging measure further demonstrated the differences shared by PA and FI. A similar finding was reported in a Canadian study in which FI-combined (the sum of the deficits in blood biomarkers and functional items) shows greater addition in the predictive utility of mortality relative to each single FI measure based on either blood biomarkers or functional terms (30). PCA is a simple dimensionality reduction technique that transforms the columns of an original dataset into a new set of features called PCs. By doing this, a large amount of the information across the original dataset is effectively compressed in fewer feature columns (i.e., the variance). Here, partially due to that PC1 captures the characteristics/information across hierarchical levels, our analysis (Table 2, Figures 2D, 3, 4) confirms that PC1 outperformed each single aging measure in terms of mortality prediction and associations with lifestyle factors. The findings suggest that aging measures at phenotypic and functional levels might be complementary (8). This indicates that integrating information across hierarchical levels may have the potential to develop better aging measures.

In addition to helping identify persons at risk, aging measures also serve as a potential endpoint for geroprotective programs. That being said, ideal aging measures should be responsive to risk factors (10). In this study, PA and FI were found to meet this criterion since they were responsive to some modifiable life style factors such as smoking status, BMI, alcohol consumption, and PAQ, which are largely consistent with previous studies (31–33). More interestingly, the new composite aging measure we developed, PC1, was strongly responsive to the same set of modifiable life style factors, highlighting its qualification as an aging measure.

Our findings have important implications in both large-scale epidemiological studies and clinical settings. First, the predictive utility of mortality risk by these aging measures (PA, FI, and PC1) suggests that we could identify vulnerable persons at risk of premature death. Together with the fact that they were responsive to modifiable life style factors, it seems that life style-targeted interventions may have the potential to slow aging and further reduce the burden of premature death. Finally, one can also apply these aging measures to examine the roles of various factors in healthy aging. Furthermore, it is promising to use these aging measures (particularly PC1) to test the effectiveness of antiaging interventions and therapies in human beings, where these aging measures serves as surrogate markers of life span. Application of aging measures is more practical and feasible in comparison to previous approaches using endpoints such as death, and the occurrence of chronic diseases, the latter requiring a long time of follow-up and high expenditures.

The present study has several strengths. First, we compared aging measures at three hierarchical levels in the same population, which is scarce in the literature. Second, the three aging measures we adopted in this study are widely recognized in the literature. We also acknowledge limitations in this study. First, the findings were based on the US population and thus may not be generalizable to other populations from different countries. Second, due to the unavailability of repeated measurements of these aging measures, we were unable to evaluate the associations between the rate of changes in aging measures and mortality risk. Third, NHANES did not collect information on exposure duration (except for smoking), which might have an impact on the results. Finally, only one aging measure at each hierarchical level was considered, in particular, only TL at the biological level. In recent years, DNA methylation age has been widely demonstrated as a promising aging measure (34–38); however, it was not available in the NHANES data. In moving forward, with more aging measures available, a more comprehensive picture of aging would be forthcoming.

## Conclusions

Our study demonstrates that both phenotypic (i.e., PA) and functional (i.e., FI) aging measures can capture mortality risk and respond to modifiable life style factors, despite their inherent differences. Furthermore, the PC1 that integrated phenotypic and functional aging measures outperforms in predicting mortality risk in comparison with each single aging measure, and strongly responds to modifiable life style factors. The findings suggest the complementary of aging measures at different hierarchical levels and underscore the need to involve multi-level information when quantifying aging. The findings also highlight the potential of life style-targeted interventions as geroprotective programs.

## Data Availability Statement

Publicly available datasets were analyzed in this study. The data can be found at the National Health and Nutrition Examination Survey website: https://www.cdc.gov/nchs/nhanes/index.htm.

## Ethics Statement

The studies involving human participants were reviewed and approved by National Center for Health Statistics Research Ethics Review Board. The patients/participants provided their written informed consent to participate in this study.

## Author Contributions

Conceptualization was proposed by XW and ZL. The methodological design and data analyses were conducted by JZ and ZL. Data collection and preparation were performed by XC, CC, and ZL. The results were interpreted by JX and LHa. The first draft of the manuscript was written by JZ, XC, LHe, and ZR. XW and ZL provided overall supervision. All authors reviewed and edited the manuscript. All authors contributed to the article, reviewed, edited the manuscript, and approved the submitted version.

## Funding

This research was supported by a grant from the National Natural Science Foundation of China (82171584), the 2020 Milstein Medical Asian American Partnership Foundation Irma and Paul Milstein Program for Senior Health project award (ZL), the Fundamental Research Funds for the Central Universities, a project from the Natural Science Foundation of Zhejiang Province (LQ21H260003), and fundings from Key Laboratory of Intelligent Preventive Medicine of Zhejiang Province (2020E10004), Leading Innovative and Entrepreneur Team Introduction Program of Zhejiang (2019R01007), Key Research and Development Program of Zhejiang Province (2020C03002), and Zhejiang University Global Partnership Fund (188170-11103). The funders had no role in the study design; data collection, analysis, or interpretation; in the writing of the report; or in the decision to submit the article for publication.

## Conflict of Interest

The authors declare that the research was conducted in the absence of any commercial or financial relationships that could be construed as a potential conflict of interest.

## Publisher's Note

All claims expressed in this article are solely those of the authors and do not necessarily represent those of their affiliated organizations, or those of the publisher, the editors and the reviewers. Any product that may be evaluated in this article, or claim that may be made by its manufacturer, is not guaranteed or endorsed by the publisher.

## References

[1] Ferrucci L Levine ME Kuo PL Simonsick EM. Time and the metrics of aging. Circ Res. (2018) 123:740–4. 10.1161/CIRCRESAHA.118.312816 30355074 PMC6205734

[2] Horvath S Raj K. DNA methylation-based biomarkers and the epigenetic clock theory of ageing. Nat Rev Genet. (2018) 19:371–84. 10.1038/s41576-018-0004-3 29643443

[3] Bell CG Lowe R Adams PD Baccarelli AA Beck S Bell JT. DNA methylation aging clocks: challenges and recommendations. Genome Biol. (2019) 20:249. 10.1186/s13059-019-1824-y 31767039 PMC6876109

[4] Liu Z Kuo PL Horvath S Crimmins E Ferrucci L Levine M. A new aging measure captures morbidity and mortality risk across diverse subpopulations from NHANES IV: a cohort study. PLoS Med. (2018) 15:e1002718. 10.1371/journal.pmed.1002718 30596641 PMC6312200

[5] Mitnitski AB Mogilner AJ Rockwood K. Accumulation of deficits as a proxy measure of aging. ScientificWorldJournal. (2001) 1:323–36. 10.1100/tsw.2001.58 12806071 PMC6084020

[6] Rockwood K Mitnitski A. Frailty in relation to the accumulation of deficits. J Gerontol A Biol Sci Med Sci. (2007) 62:722–7. 10.1093/gerona/62.7.722 17634318

[7] Rockwood K Song X MacKnight C Bergman H Hogan DB McDowell I. A global clinical measure of fitness and frailty in elderly people. CMAJ. (2005) 173:489–95. 10.1503/cmaj.050051 16129869 PMC1188185

[8] Li X Ploner A Wang Y Magnusson PK Reynolds C Finkel D. Longitudinal trajectories, correlations and mortality associations of nine biological ages across 20-years follow-up. Elife. (2020) 9:e51507. 10.7554/eLife.51507 32041686 PMC7012595

[9] Lehallier B Gate D Schaum N Nanasi T Lee SE Yousef H. Undulating changes in human plasma proteome profiles across the lifespan. Nat Med. (2019) 25:1843–50. 10.1038/s41591-019-0673-2 31806903 PMC7062043

[10] Justice JN Kritchevsky SB. Putting epigenetic biomarkers to the test for clinical trials. Elife. (2020) 9:e58592. 10.7554/eLife.58592 32515735 PMC7282804

[11] Simpson RJ Lowder TW Spielmann G Bigley AB LaVoy EC Kunz H. Exercise and the aging immune system. Ageing Res Rev. (2012) 11:404–20. 10.1016/j.arr.2012.03.003 22465452

[12] Brown JD Alipour-Haris G Pahor M Manini TM. Association between a deficit accumulation frailty index and mobility outcomes in older adults: secondary analysis of the lifestyle interventions and independence for elders (LIFE) study. J Clin Med. (2020) 9:3757. 10.3390/jcm9113757 33266358 PMC7700674

[13] Huang CH Umegaki H Makino T Uemura K Hayashi T Kitada T. Effect of various exercises on frailty among older adults with subjective cognitive concerns: a randomised controlled trial. Age Ageing. (2020) 49:1011–9. 10.1093/ageing/afaa086 32520984

[14] Cawthon RM. Telomere measurement by quantitative PCR. Nucleic Acids Res. (2002) 30:e47. 10.1093/nar/30.10.e47 12000852 PMC115301

[15] Needham BL Adler N Gregorich S Rehkopf D Lin J Blackburn EH. Socioeconomic status, health behavior, and leukocyte telomere length in the national health and nutrition examination survey, 1999-2002. Soc Sci Med. (2013) 85:1–8. 10.1016/j.socscimed.2013.02.023 23540359 PMC3666871

[16] Levine ME Lu AT Quach A Chen BH Assimes TL Bandinelli S. An epigenetic biomarker of aging for lifespan and healthspan. Aging. (2018) 10:573–91. 10.18632/aging.101414 29676998 PMC5940111

[17] Blodgett JM Theou O Howlett SE Rockwood K. A frailty index from common clinical and laboratory tests predicts increased risk of death across the life course. Geroscience. (2017) 39:447–55. 10.1007/s11357-017-9993-7 28866737 PMC5636769

[18] National Center for Health Statistics. Office of Analysis and Epidemiology, Public-use Linked Mortality File. Hyattsville, MD (2015). Available onlinev at: https://www.cdc.gov/nchs/data-linkage/mortality-public.htm.

[19] Guenther PM Kirkpatrick SI Reedy J Krebs-Smith SM Buckman DW Dodd KW. The healthy eating index-2010 is a valid and reliable measure of diet quality according to the 2010 dietary guidelines for Americans. J Nutr. (2014) 144:399–407. 10.3945/jn.113.183079 24453128 PMC3927552

[20] Kamarudin AN Cox T Kolamunnage-Dona R. Time-dependent ROC curve analysis in medical research: current methods and applications. BMC Med Res Methodol. (2017) 17:53. 10.1186/s12874-017-0332-6 28388943 PMC5384160

[21] Pencina MJ D'Agostino RB D'Agostino RB Jr Vasan RS. Evaluating the added predictive ability of a new marker: from area under the ROC curve to reclassification and beyond. Stat Med. (2008) 27:157–72; discussion 207–12. 10.1002/sim.2929 17569110

[22] Kojima G Iliffe S Walters K. Frailty index as a predictor of mortality: a systematic review and meta-analysis. Age Ageing. (2018) 47:193–200. 10.1093/ageing/afx162 29040347

[23] Fan J Yu C Guo Y Bian Z Sun Z Yang L. Frailty index and all-cause and cause-specific mortality in Chinese adults: a prospective cohort study. Lancet Public Health. (2020) 5:e650–60. 10.1016/S2468-2667(20)30113-4 33271078 PMC7708389

[24] Liu Z Chen X Gill TM Ma C Crimmins EM Levine ME. Associations of genetics, behaviors, and life course circumstances with a novel aging and healthspan measure: evidence from the health and retirement study. PLoS Med. (2019) 16:e1002827. 10.1371/journal.pmed.1002827 31211779 PMC6581243

[25] Belsky DW Moffitt TE Cohen AA Corcoran DL Levine ME Prinz JA. Eleven telomere, epigenetic clock, and biomarker-composite quantifications of biological aging: do they measure the same thing? Am J Epidemiol. (2018) 187:1220–30. 10.1093/aje/kwx346 29149257 PMC6248475

[26] Wang Q Zhan Y Pedersen NL Fang F Hägg S. Telomere length and all-cause mortality: a meta-analysis. Ageing Res Rev. (2018) 48:11–20. 10.1016/j.arr.2018.09.002 30254001

[27] Hastings WJ Shalev I Belsky DW. Comparability of biological aging measures in the national health and nutrition examination study, 1999-2002. Psychoneuroendocrinology. (2019) 106:171–8. 10.1016/j.psyneuen.2019.03.012 30999227 PMC6599717

[28] Cawthon RM Smith KR O'Brien E Sivatchenko A Kerber RA. Association between telomere length in blood and mortality in people aged 60 years or older. Lancet. (2003) 361:393–5. 10.1016/S0140-6736(03)12384-7 12573379

[29] Sanders JL Newman AB. Telomere length in epidemiology: a biomarker of aging, age-related disease, both, or neither? Epidemiol Rev. (2013) 35:112–31. 10.1093/epirev/mxs008 23302541 PMC4707879

[30] Howlett SE Rockwood MR Mitnitski A Rockwood K. Standard laboratory tests to identify older adults at increased risk of death. BMC Med. (2014) 12:171. 10.1186/s12916-014-0171-9 25288274 PMC4190374

[31] Kresovich JK Garval EL Martinez Lopez AM Xu Z Niehoff NM White AJ. Associations of body composition and physical activity level with multiple measures of epigenetic age acceleration. Am J Epidemiol. (2021) 190:984–93. 10.1093/aje/kwaa251 33693587 PMC8168202

[32] Peng H Gao W Cao W Lv J Yu C Wu T. Combined healthy lifestyle score and risk of epigenetic aging: a discordant monozygotic twin study. Aging. (2021) 13:14039–52. 10.18632/aging.203022 34032609 PMC8202852

[33] Ng TP Zhong X Gao Q Gwee X Chua DQL Larbi A. Socio-Environmental, lifestyle, behavioural, and psychological determinants of biological ageing: the singapore longitudinal ageing study. Gerontology. (2020) 66:603–13. 10.1159/000511211 33197920

[34] Chen BH Marioni RE Colicino E Peters MJ Ward-Caviness CK Tsai PC. DNA methylation-based measures of biological age: meta-analysis predicting time to death. Aging. (2016) 8:1844–65. 10.18632/aging.101020 27690265 PMC5076441

[35] Marioni RE Shah S McRae AF Chen BH Colicino E Harris SE. DNA methylation age of blood predicts all-cause mortality in later life. Genome Biol. (2015) 16:25. 10.1186/s13059-015-0584-6 25633388 PMC4350614

[36] Levine ME Lu AT Bennett DA Horvath S. Epigenetic age of the pre-frontal cortex is associated with neuritic plaques, amyloid load, and Alzheimer's disease related cognitive functioning. Aging. (2015) 7:1198–211. 10.18632/aging.100864 26684672 PMC4712342

[37] Martin-Herranz DE Aref-Eshghi E Bonder MJ Stubbs TM Choufani S Weksberg R. Screening for genes that accelerate the epigenetic aging clock in humans reveals a role for the H3K36 methyltransferase NSD1. Genome Biol. (2019) 20:146. 10.1186/s13059-019-1753-9 31409373 PMC6693144

[38] Zheng Y Joyce BT Colicino E Liu L Zhang W Dai Q. Blood epigenetic age may predict cancer incidence and mortality. EBioMedicine. (2016) 5:68–73. 10.1016/j.ebiom.2016.02.008 27077113 PMC4816845

